# MEG Activity in Visual and Auditory Cortices Represents Acoustic Speech-Related Information during Silent Lip Reading

**DOI:** 10.1523/ENEURO.0209-22.2022

**Published:** 2022-06-27

**Authors:** Felix Bröhl, Anne Keitel, Christoph Kayser

**Affiliations:** 1 Department for Cognitive Neuroscience, Faculty of Biology, Bielefeld University, Bielefeld 33615, Germany; 2 Psychology, University of Dundee, Dundee DD1 4HN, United Kingdom

**Keywords:** audiovisual, language, lip reading, MEG, speech entrainment, speech tracking

## Abstract

Speech is an intrinsically multisensory signal, and seeing the speaker’s lips forms a cornerstone of communication in acoustically impoverished environments. Still, it remains unclear how the brain exploits visual speech for comprehension. Previous work debated whether lip signals are mainly processed along the auditory pathways or whether the visual system directly implements speech-related processes. To probe this, we systematically characterized dynamic representations of multiple acoustic and visual speech-derived features in source localized MEG recordings that were obtained while participants listened to speech or viewed silent speech. Using a mutual-information framework we provide a comprehensive assessment of how well temporal and occipital cortices reflect the physically presented signals and unique aspects of acoustic features that were physically absent but may be critical for comprehension. Our results demonstrate that both cortices feature a functionally specific form of multisensory restoration: during lip reading, they reflect unheard acoustic features, independent of co-existing representations of the visible lip movements. This restoration emphasizes the unheard pitch signature in occipital cortex and the speech envelope in temporal cortex and is predictive of lip-reading performance. These findings suggest that when seeing the speaker’s lips, the brain engages both visual and auditory pathways to support comprehension by exploiting multisensory correspondences between lip movements and spectro-temporal acoustic cues.

## Significance Statement

Lip reading is central for speech comprehension in acoustically impoverished environments. Recent studies show that the auditory and visual cortex can represent acoustic speech features from purely visual speech. It is still unclear, however, what information is represented in these cortices and whether this phenomenon is related to lip-reading comprehension. Using a comprehensive conditional mutual information (MI) analysis applied to magnetoencephalographic data, we demonstrate that signatures of acoustic speech arise in both cortices in parallel, even when discounting for the physically presented stimulus. In addition, the auditory but not the visual cortex activity was related to successful lip reading across participants.

## Introduction

Speech is an intrinsically multisensory stimulus that can be conveyed via acoustic and visual signals. It remains debated how the brain exploits the information derived from visual speech (Calvert et al., 1997; Grant and Seitz, 2000; Calvert and Campbell, 2003; Besle et al., 2008). One view is that the visual system directly contributes to establishing speech representations (Bernstein et al., 2011; O’Sullivan et al., 2017; Ozker et al., 2018), as oro-facial movements provide temporal information that can be predictive of concurrent acoustic signals and allow mapping visual cues onto phonological representations (Campbell, 2008; Lazard and Giraud, 2017). The visual cortex tracks dynamic lip signals (Park et al., 2016) and, as suggested recently, may also directly “restore” the acoustic envelope of the visually presented speech (Hauswald et al., 2018; Suess et al., 2022). Another view is that visual speech is mainly represented in regions of the auditory pathways, possibly exploiting speech-specific processes of this system. Along this line, a recent study suggested that the early auditory cortex may also be capable of reflecting the unheard acoustic envelope of a spoken narrative (Bourguignon et al., 2020). Importantly, the evidence that visual speech is reflected along both auditory and the visual pathways may not be mutually exclusive, as both may contribute to a supramodal frame of reference for speech (Arnal et al., 2009; Rauschecker, 2012).

To probe the respective involvement of visual and auditory cortices in representing visual speech, many previous studies presented syllables or isolated words as stimuli (Calvert et al., 1997; Calvert and Campbell, 2003; Pekkola et al., 2005). However, these results come short of how they translate to continuous or natural speech. Furthermore, many studies did not probe a direct link to behavioral performance, leaving it unclear whether potential cerebral representations derived from visual speech are behaviorally relevant (Ludman et al., 2000; Bourguignon et al., 2020; Mégevand et al., 2020). The latter can be particularly challenging given that pure lip-reading performance for everyday speech is often low (Grant and Seitz, 2000; Altieri et al., 2011).

The present study rests on the assumption that probing the roles of visual and auditory cortices in representing visual speech requires data from a paradigm based on continuous speech with carefully controlled levels of lip-reading performance. In previous work we established such a paradigm and collected MEG data from participants during a word recognition task based on syntactically similar sentences that were presented either purely acoustically or purely visually. In the auditory condition participants were presented with the acoustic signal embedded in background noise, while in the visual condition they watched the muted speaker. The individual sentences were constructed from a closed-set of linguistic items with a common syntactic structure, similar to matrix-sentences used in standardized hearing assessment (Hagerman, 1982; Kollmeier et al., 2015). With this we achieved a comparable level of word recognition performance during auditory-only and visual-only conditions and verified that this dataset allows linking neural representations of lexical information and speech dynamics to behavior (Keitel et al., 2018, 2020).

We leverage this paradigm to probe the roles of visual and auditory pathways in representing visual speech and facilitating lip-reading performance. This led us to formulate the two following questions. First, whether representations of restored (e.g., unheard acoustic) features are independent of those physically present (e.g., lip movements). Second, we asked whether these representations of restored features are tied to word recognition performance. To be able to compare cerebral signatures of visual and acoustic speech-derived features, we rooted this analysis on the following systematic assessment: we quantified how well source-localized MEG signals track multiple acoustic and visual speech-derived features independently of each other, both when these features are physically present (e.g., the lip contour when watching the speaker) or absent (e.g., the pitch contour when watching the speaker). We focused this analysis on two regions of interest (ROIs) centered on early auditory and visual cortices, which previous studies have implied in supporting lip reading (Park et al., 2016; Hauswald et al., 2018; Bourguignon et al., 2020). The analysis was based on a mutual information (MI) approach that has been used in previous work to probe dynamic speech representations and which is well suited to address the statistical dependency between multiple variables (Keitel et al., 2018; Daube et al., 2019). Our results show that both occipital and temporal regions reflect unheard acoustic speech-derived features independently of the physically present lip movements. This “restoration” of acoustic information in the temporal, but not the occipital, cortex is predictive of word recognition performance across participants.

## Materials and Methods

The data analyzed in this study has been collected and analyzed in previous studies (Keitel et al., 2018, 2020). The analyses conducted here pose new questions and provide novel results beyond the previous work.

### Participants and data acquisition

Data were collected from 20 native British-English speaking participants (nine female, age 23.6 ± 5.8 years mean ± SD). Because of prominent environmental artefacts in the MEG recordings, data from two participants were excluded from further analysis. Thus, the analyzed data were from 18 participants (seven female, age 24 ± 6.0 years mean ± SD). All participants were screened to exclude hearing impairment before data collection using the quick hearing check questionnaire (Koike et al., 1994), had normal or corrected-to-normal vision and were all right-handed (Oldfield, 1971). All participants provided written informed consent and received monetary compensation of 10 £/h. The experiment was approved by the College of Science and Engineering, University of Glasgow (approval number 300140078) and conducted in compliance with the Declaration of Helsinki.

MEG data were collected using a 248-magnetometer whole-head MEG system (MAGNES 3600 WH, 4-D Neuroimaging) with a sample rate of 1 kHz. Head positions were measured at the beginning and end of each run, using five coils placed on the participants’ heads. Coil positions were co-digitized with the participant’s head-shape (FASTRAK, Polhemus Inc.). Participants were seated in an upright position in front of a screen. Visual stimuli were displayed with a DLP projector at 25 frames per second (fps), a resolution of 1280 × 720 pixels, and covered a visual field of 25 × 19 degrees. Acoustic stimuli were transmitted binaurally through plastic earpieces and 370-cm-long plastic tubes connected to a sound pressure transducer and were presented in stereo at a sampling rate of 22,050 Hz.

### Stimulus material

The stimulus material comprised two structurally equivalent sets of 90 unique closed-set English sentences. Specifically, along the idea of matrix-style sentences using in standardized hearing assessment (Hagerman, 1982; Kollmeier et al., 2015), each sentence was constructed with the same sequence of linguistic elements, the order of which can be described with the following pattern [filler phrase, time phrase, name, verb, numeral, adjective, noun]. One such sentence for example was “I forgot to mention (filler phrase), last Thursday morning (time phrase) Mary (name) obtained (verb) four (numeral) beautiful (adjective) journals (noun).” For each element, a list of 18 different options was created and sentences were constructed so that each single element was repeated ten times. Sentence elements were randomly combined within each set of 90 sentences. This procedure yielded 180 structurally similar but distinct sentences. To measure word recognition performance for each sentence, a target word was defined in each sentence: either the adjective (first set of sentences) or the numeral (second set). Sentences lasted on average 5.4 ± 0.4 s (mean ± SD, ranging from 4.6 to 6.5 s) and lasted a total of ∼22 min. The speech material was spoken by a male British actor, who was tasked to speak clearly and naturally and to move as little as possible while speaking to assure that the lips center stayed at the same place in each video frame. Audiovisual recordings were gathered with a high-performance camcorder (Sony PMW-EX1) and an external microphone in a sound attenuating booth.

Participants were presented with audio-only (A-only), audiovisual (AV) or visual-only (V-only) speech material in three conditions (Keitel et al., 2020). However, for the present analysis we only focus on the A-only and V-only conditions, as in these, one can best dissociate visual-related and auditory-related speech representations given that only one physical stimulus was present. Furthermore, during the AV condition word recognition performance was near-ceiling (Keitel et al., 2020), making it difficult to link cerebral and behavioral data. Because performance would have been at ceiling with clear speech in the A-only condition, the acoustic speech was embedded in environmental noise. This noise for each trial was generated by randomly selecting 50 individual sounds from a set of sounds recorded from natural, everyday sources or scenes (e.g., car horns, talking people, traffic). These sounds were then added together to create a distracting noise scene for the duration of each trial. For each participant the individual noise level was further adjusted, as described previously (Keitel et al., 2020). This resulted in an average performance of ∼70% correct for both A-only and V-only conditions and allowed us to dissociate between correct and incorrect word recognition.

### Experimental design

Each participant was presented with each of the 180 sentences in three conditions (A-only, V-only, and AV). The order of the conditions was fixed for all participants as A-only, AV and then V-only. This order exposed the participants to the stimuli twice before the lip-reading task, which helped to increase performance and render it comparable to the A-only task. Each condition was divided into four blocks of 45 sentences each, with two blocks being “adjective” and two “number” blocks. For each participant, the order of sentences within each block was randomized. The first sentence of each block was a “dummy” trial that was subsequently excluded from analysis. During each trial, participants either fixated a dot (in A condition) or a small cross overlaid onto the mouth of the speaker’s face (in V condition). In the A condition, each sentence was presented as the respective audio recording, i.e., the spoken sentence, together with the background noise. In the V condition, only the video of the speaker’s face was presented clearly and no sound was present. After each trial, four words were presented as response options (either four adjectives or four written numbers) on the screen and participants had to indicate using a button press which word they had perceived. Intertrial intervals were set to last about 2 s.

### Preprocessing of stimulus material

From the stimulus material, we extracted the following auditory and visual features. Based on previous literature that demonstrated robust encoding of the amplitude envelope, its temporal derivative and the fundamental frequency of speech, we derived these features from the acoustic speech recordings (Oganian and Chang, 2019; Teoh et al., 2019; Bröhl and Kayser, 2021). To derive the broadband envelope, we filtered the acoustic waveform into 12 logarithmically spaced bands between 0.1 and 10 kHz (zero-phase third order Butterworth filter with boundaries: 0.1, 0.18, 0.3, 0.46, 0.68, 0.98, 1.39, 2.0, 2.73, 3.79, 5.25, 7.25, 10 kHz) and subsequently took the absolute value of the Hilbert transform for each band. The broadband amplitude envelope (hereon termed aud env) was then derived by taking the average across all 12 band-limited envelopes and was subsequently down-sampled to 50 Hz. We computed the slope of this broadband envelope (hereon termed aud slope) by taking its first derivative. To characterize the pitch contour, we extracted the fundamental frequency (hereon termed aud pitch) over time using the Praat software (“to Pitch” method with predefined parameters; Boersma and van Heuven, 2001). This was done using the original acoustic waveform at a sampling rate of 22,050 Hz. The resulting pitch contour was again down sampled to 50 Hz. All three acoustic features together are labeled AudFeat in the following.

In a similar fashion, we derived the horizontal opening of the lips, the area covered by the lip opening, and its derivative from the video recordings. The lips were detected based on the color of the lips in the video material using a custom-made algorithm. From these we determined the contour of the lip opening based on luminance values and deriving connected components from these (Giordano et al., 2017). The results were visually inspected to ensure accurate tracking of the lips. From this segmentation of the lip opening, we derived the total opening (in pixels; hereon termed lip area) and estimates of the respective diameters along the horizontal axis (hereon termed lip width): these were defined between the outermost points along the horizontal axis. These signals were initially sampled at the video rate of 25 fps. Similar to the auditory features, we computed the slope of the lip area (hereon termed lip slope). The time series of these visual features were then linearly interpolated to a sample rate of 50 Hz. Because the horizontal and vertical mouth openings are partially correlated with each other and with the total mouth opening, we selected the total area and the horizontal width as signals of interest, as the latter is specifically informative about the acoustic formant structure (Plass et al., 2020). We grouped the total lip area, its temporal derivative, and the lip-width as signatures of lip features (LipFeat), which are of the same dimensionality as the acoustic features (AudFeat) described above.

For comparison with previous studies (Chandrasekaran et al., 2009; Park et al., 2016; Giordano et al., 2017; Hauswald et al., 2018) we quantified the power spectra of these features and their cross-coherences using MATLAB’s ‘pwelch’ and ‘mscoher’ functions using a window length of 1 s with 50% overlap and otherwise predefined parameters. The resulting spectra were log transformed and averaged across sentences. To visualize the cross-coherences we first obtained key frequency ranges from our main results (Fig. 3) and averaged the coherences within two ranges of interest (0.5–1 and 1–3 Hz).

### MEG preprocessing

Preprocessing of MEG data was conducted using custom MATLAB scripts and the FieldTrip toolbox (Oostenveld et al., 2011). Each experimental block was processed separately. Individual trials were extracted from continuous data starting 2 s before sound onset and until 10 s after sound onset. The MEG data were denoised using a reference signal. Known faulty channels (*N* = 7) were removed. Trials with SQUID jumps (3.5% of trials) were detected and removed using FieldTrip procedures with a cutoff z-value of 30. Data were bandpass filtered between 0.2 and 150 Hz using a zero-phase fourth order Butterworth filter and subsequently down sampled to 300 Hz before further artifact rejection. Data were visually inspected to find noisy channels (4.37 ± 3.38 on average across blocks and participants) and trials (0.66 ± 1.03 on average across blocks and participants). Noise cleaning was performed using independent component analysis with 30 principal components (2.5 components removed on average). Data were then bandpass filtered between 0.8 and 30 Hz using a zero-phase third order Butterworth filter and further down sampled to 50 Hz for subsequent analysis.

### MEG source reconstruction

Source reconstruction was performed using Fieldtrip, SPM8, and the Freesurfer toolbox based on T1-weighted structural magnetic resonance images (MRIs) for each participant. These were co-registered to the MEG coordinate system using a semi-automatic procedure (Gross et al., 2013; Keitel et al., 2017). MRIs were then segmented and linearly normalized to a template brain (MNI space). We projected sensor-level time series into source space using a frequency-specific linear constraint minimum variance (LCMV) beamformer (Van Veen et al., 1997) with a regularization parameter of 7% and optimal dipole orientation (singular value decomposition method). The grid points had a spacing of 6 mm, thus resulting in 12,337 points. For whole-brain analyses, a subset of grid points corresponding to cortical gray matter regions only was selected (using the AAL atlas, Tzourio-Mazoyer et al., 2002), yielding 6490 points in total. Within these we defined temporal and occipital ROIs based on the brainnetome atlas (Yu et al., 2011). The individual ROIs were chosen based on previous studies that demonstrate the encoding of acoustic and visual speech features in occipital and superior temporal regions (Giordano et al., 2017; Di Liberto et al., 2018; Teng et al., 2018; Keitel et al., 2020). As temporal ROI, we included Brodmann area 41/42, caudal area 22 (A22c), rostral area 22 (A22r), and TE1.0 and TE1.2. As occipital ROI, we defined the middle occipital gyrus (mOccG), occipital polar gyrus (OPC), inferior occipital gyrus (iOccG), and the medial superior occipital gyrus (msOccG).

### MEG analysis

**Figure 1.**
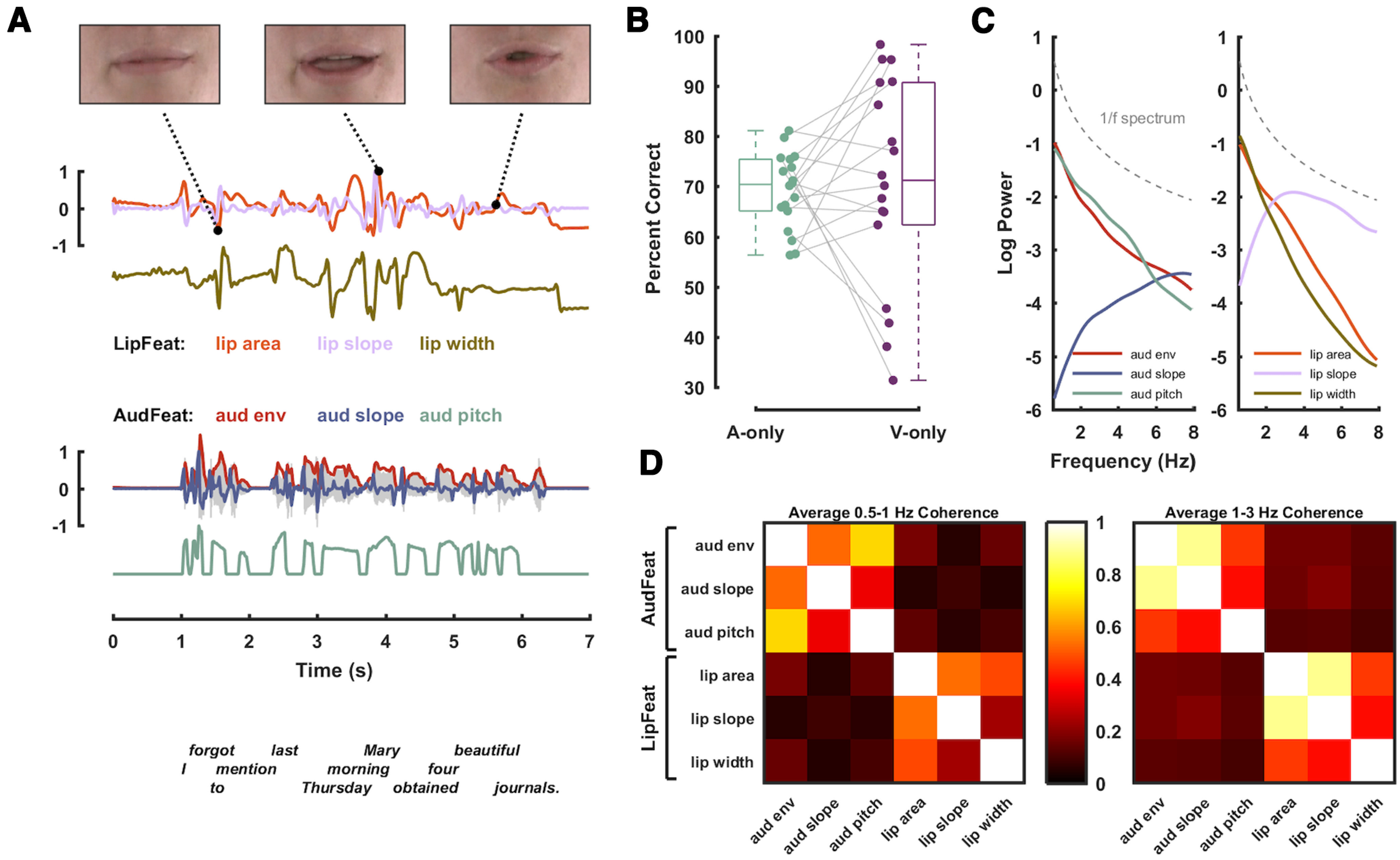
Stimulus material and experimental methodology. Acoustic and visual features were extracted from audiovisual speech material and were used to quantify their cerebral tracking during audio-only and visual-only presentations. ***A***, The stimulus material consisted of 180 audiovisual recordings of a trained actor speaking individual English sentences. For visualization, here only the mouth is shown, but participants were presented with the entire face. From the video recordings, we extracted three features describing the dynamics of the lip aperture: the area of lip opening (lip area), its slope (lip slope), and the width of lip opening (lip width); collectively termed LipFeat. From the audio waveform, we extracted three acoustic features: the broadband envelope (aud env), its slope (aud slope), and a measure of dominant pitch (aud pitch), collectively termed AudFeat. ***B***, Trial-averaged percent correctly (PC) reported target words in auditory (A-only) and visual-only (V-only) conditions, with dots representing individual participants. ***C***, Logarithmic power spectra for individual stimulus features. For reference, a 1/f spectrum is shown as a dashed gray line. ***D***, Coherence between pairs of features averaged within two predefined frequency bands (0.5–1 Hz left; 1–3 Hz right; for details, see Materials and Methods).

The questions outlined in the introduction require quantifying how well the source reconstructed MEG data reflect the visual and or acoustic features. For this we relied on a previously established and validated MI framework (Ince et al., 2017). The analysis relies on the notion that a significant temporal relation between a cerebral signal and sensory features is indicating the cerebral encoding (or tracking) of the respective features in temporally entrained brain activity (Park et al., 2016; Keitel et al., 2018; Bröhl and Kayser, 2021). In the following we use the term “tracking” when referring to such putative cerebral representations characterized using MI (Obleser and Kayser, 2019). To quantify the tracking of a given stimulus feature, or of a feature group, we concatenated the trial-wise MEG data and features along the time dimension and filtered these (using third order Butterworth IIR filters) into typical frequency bands used to study dynamic speech encoding: 0.5–1, 1–3, 2–4, 3–6, and 4–8 Hz (and 0.5–8 Hz). These were chosen to cover the typical modulation spectra of these features (Fig. 1*C*,*D*) and similar to previous work (Etard and Reichenbach, 2019; van Bree et al., 2020; Bröhl and Kayser, 2021; Zuk et al., 2021). The first 500 ms of each sentence were discarded to remove the influence of the transient sound-onset response. To compute the MI between filtered MEG and stimulus features, we relied on a complex-valued representation of each signal, which allowed us to include both the amplitude and phase information in the analysis: we first derived the analytic signal of both the MEG and stimulus feature(s) using the Hilbert transform and then calculated the MI using the Gaussian copula approach including the real and imaginary part of the Hilbert signals (Ince et al., 2017; Daube et al., 2019).

**Figure 2.**
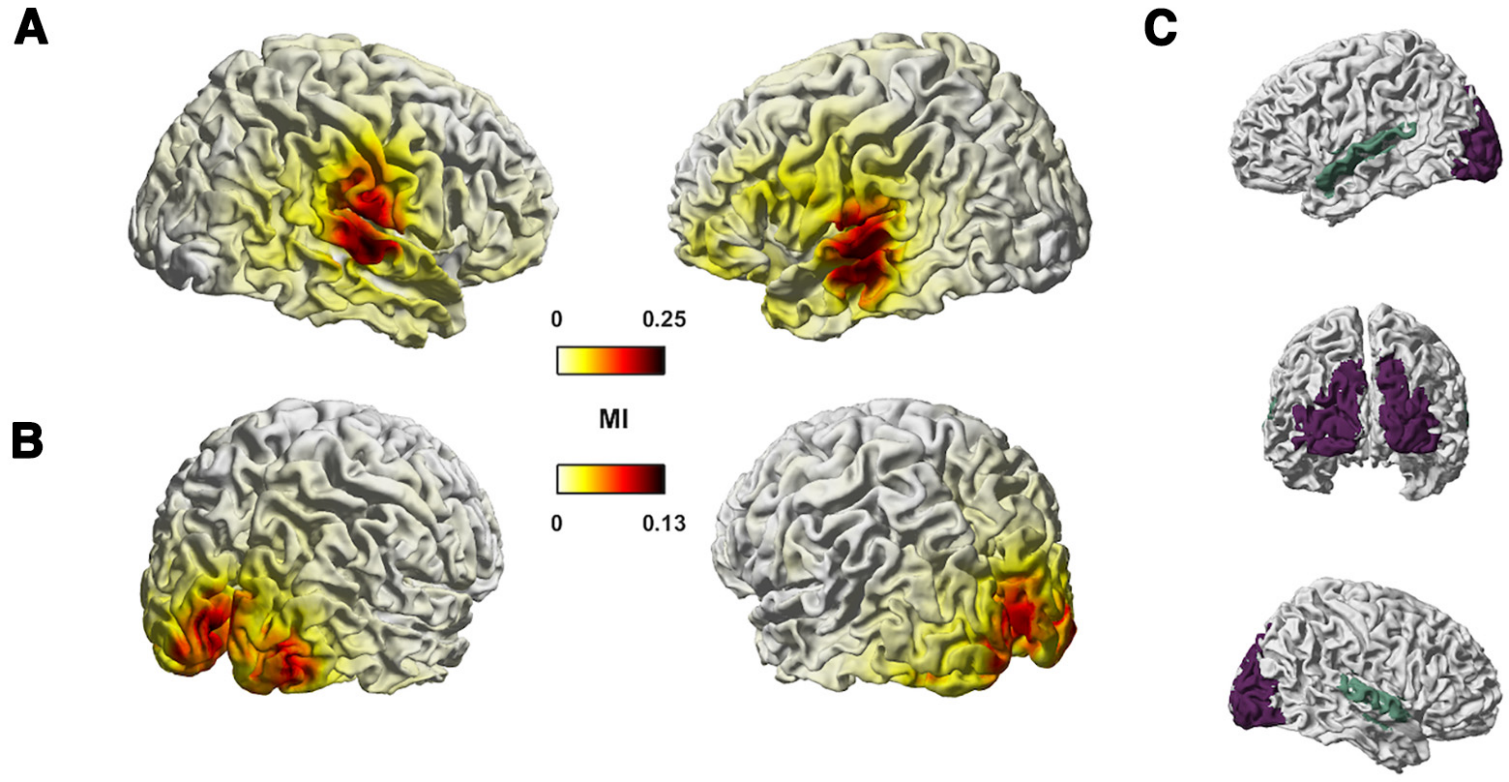
Tracking of auditory and visual features in MEG source space. The figure shows group-level median MI values for auditory (AudFeat; ***A***) and lip features (LipFeat; ***B***) in the frequency range from 0.5 to 8 Hz (*n* = 18 participants). ***C***, Colored shading indicates ROIs: temporal region in mint includes Brodmann area 41/42, caudal area 22 (A22c), rostral area 22 (A22r), and TE1.0 and TE1.2; occipital region in purple includes middle occipital gyrus (mOccG), occipital polar gyrus (OPC), inferior occipital gyrus (iOccG), and medial superior occipital gyrus (msOccG). Unit for MI is in bits.

**Figure 3.**
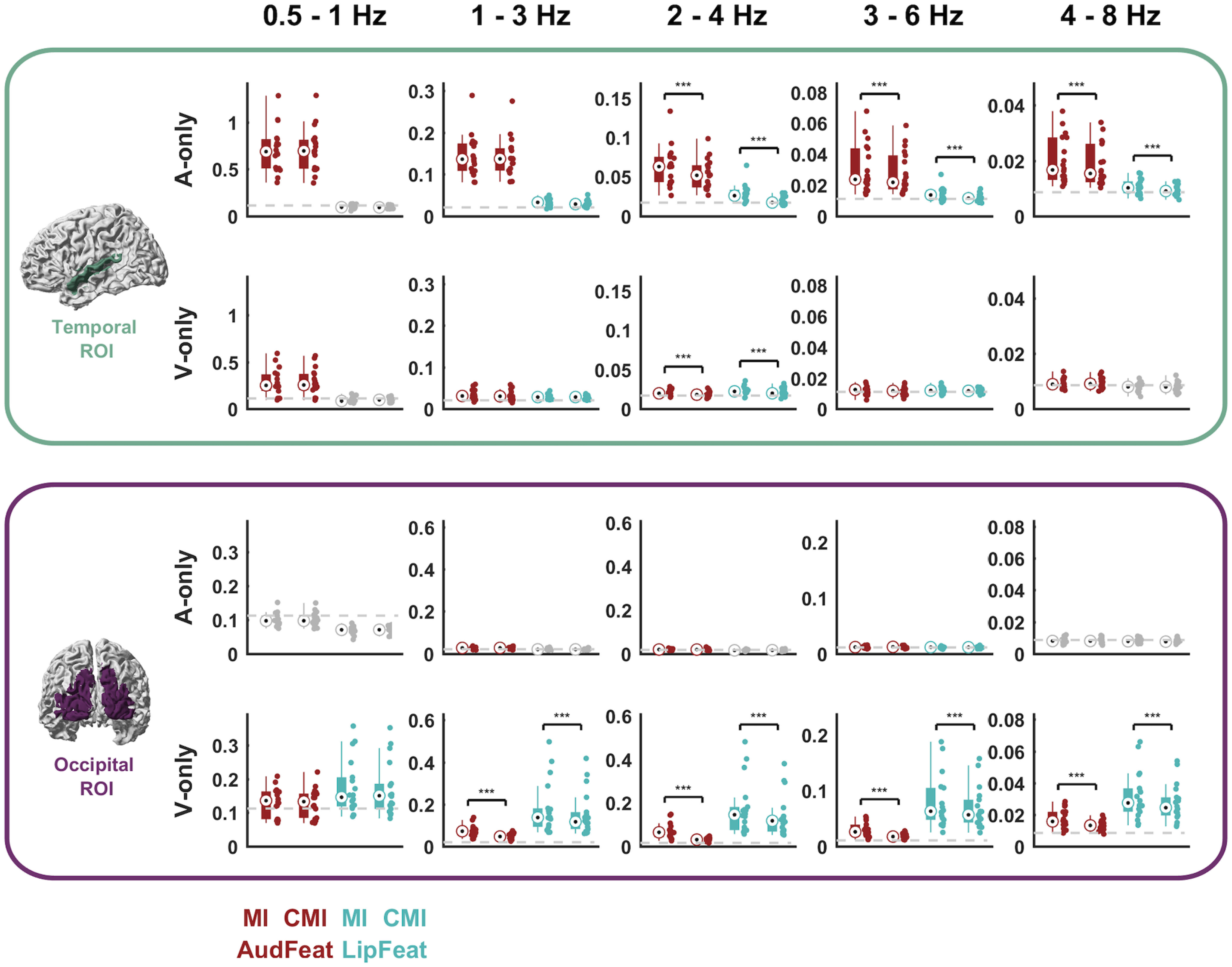
Feature tracking across ROIs and conditions. For both conditions (A-only and V-only) and ROIs (temporal and occipital) the figure illustrates the strength of feature tracking for presented and physically not-present features (MI values) and the strength of tracking after partialling out the respective other feature group (CMI values). Each panel depicts (from left to right) the MI for AudFeat, the CMI for AudFeat partialling out LipFeat, the MI for LipFeat, and the CMI for LipFeat partialling out AudFeat. Dots represent individual participants (*n* = 18). Bars indicate the median, 25th and 75th percentiles. The gray dashed line indicates the 99th percentile of the frequency-specific randomized maximum distribution correcting for all other dimensions. Conditions below a group-level significance threshold of 0.01 are greyed out. Brackets with asterisks indicate significant differences between MI and CMI values, based on a Wilcoxon signed-rank test (*p < 0.01, **p < 0.005, ***p < 0.001). Units for MI and CMI are in bits.

**Figure 4.**
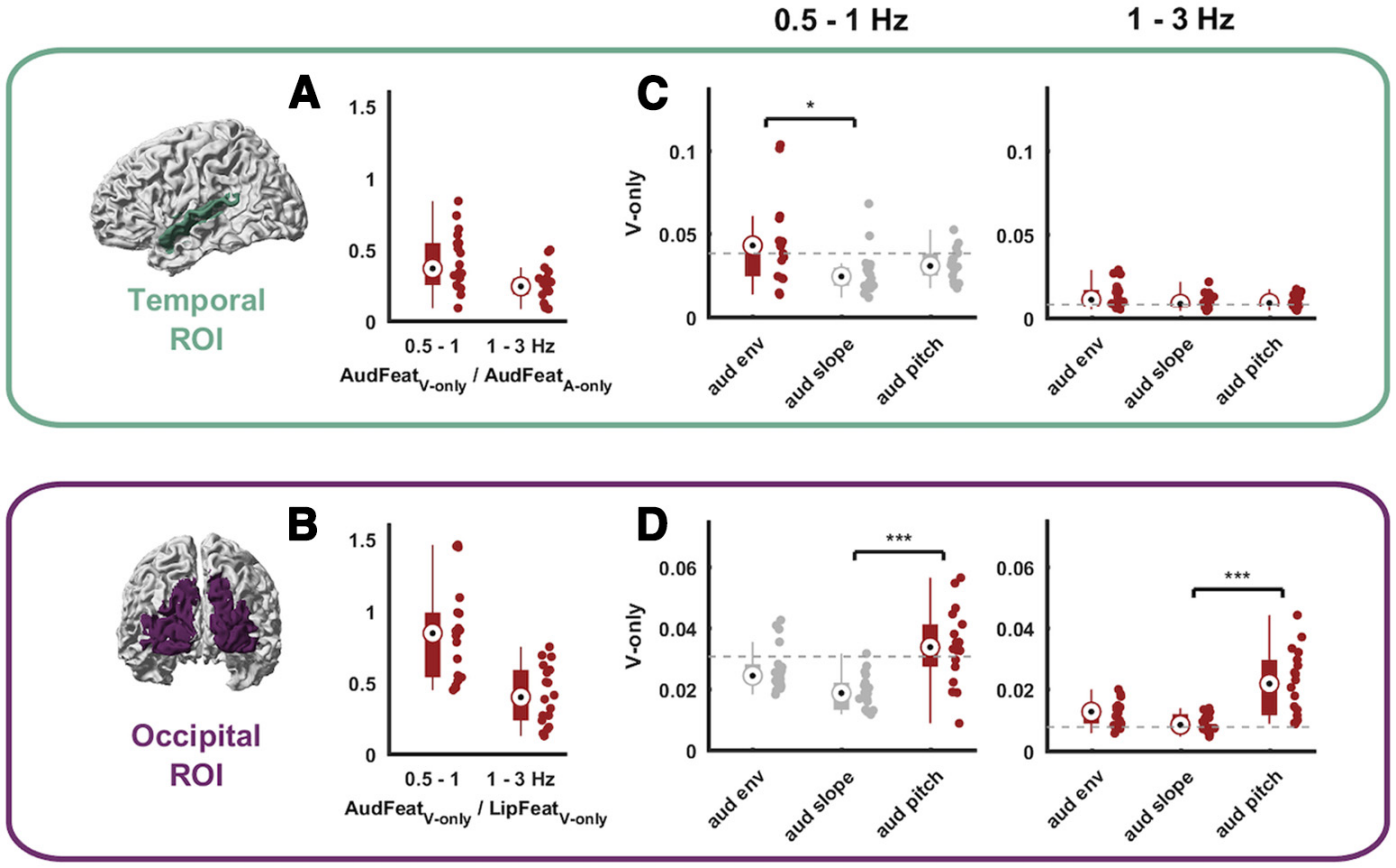
Modality dominance and tracking of individual auditory features during lip reading. ***A***, ***B***, Comparison of the tracking of unheard AudFeat over the tracking of the modality-preferred sensory input in each ROI (i.e., AudFeat during A-only trials in the temporal ROI; LipFeat during V-only trials in the occipital ROI). ***C***, ***D***, Tracking of individual auditory features during V-only trials conditioned on all other auditory and lip features in temporal (***C***) and occipital (***D***) ROIs. Brackets with asterisks indicate levels of significance from one-way Kruskal–Wallis rank test with *post hoc* Tukey–Kramer testing (*p < 0.01, **p < 0.005, ***p < 0.001). Dots represent individual data points. Bars indicate the median, 25th and 75th percentiles. The gray dashed line indicates the 99th percentile of the frequency-specific randomized maximum distribution correction for all other features. Units in ***A***, ***B*** are a ratio; in ***C*** and ***D***, units are in bits.

**Figure 5.**
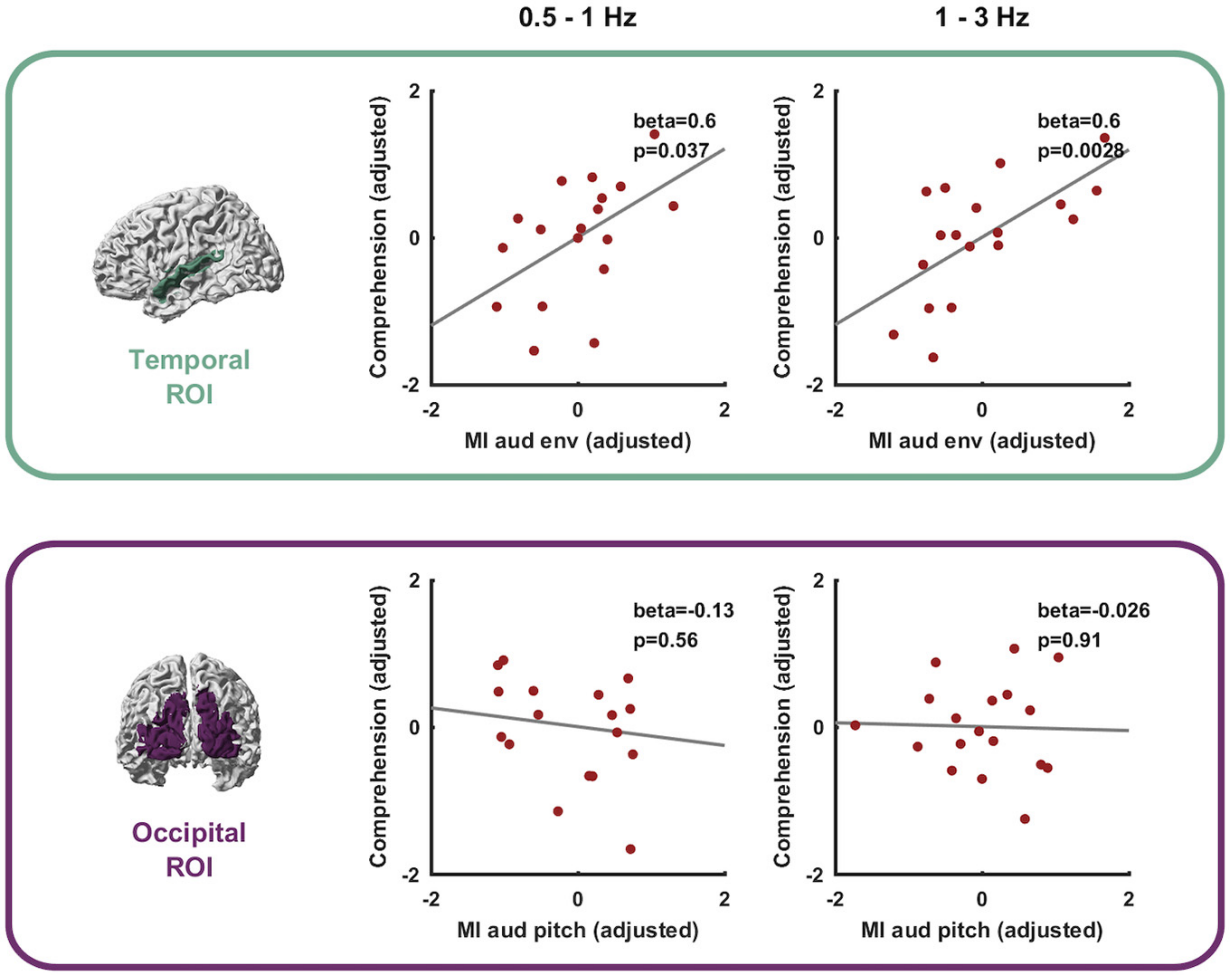
Association between lip-reading performance and tracking of auditory features. Across participants the tracking of aud env during V-only trials in the temporal ROI but not the tracking of aud pitch in the occipital ROI was significantly associated with word recognition performance (PC). Graphs show partial residual plots, dots represent individual data points and the line indicates the linear fit to the target variable from the full regression model.

In a first step, we used this framework to visualize the tracking of AudFeat and LipFeat within the entire source space (Fig. 2*A*,*B*). This was mainly done to assert that the predefined ROIs used for the subsequent analysis indeed covered the relevant tracking of these features. This analysis relied on a frequency range from 0.5 to 8 Hz and a range of stimulus-to-brain lags from 60 to 140 ms after stimulus onset. As a second step, we then quantified the tracking of auditory or visual features and their dependencies specifically within these ROIs and individual frequency bands (Figs. 3-5). To facilitate these analyses, we first determined the optimal lags for each feature, ROI and frequency band, given that the encoding latencies may differ between features and regions (Giordano et al., 2017). For this, we determined at the group-level and for each set of features (i.e., AudFeat and LipFeat) and for each ROI and frequency band the respective lag yielding the largest group-level MI value (across participants and both A-only and V-only trials). This was done by computing the MI between each set of features and the MEG in a range of lags between 0 and 500 ms in 20-ms steps. For the subsequent analyses, we used these optimal lags and computed averaged MI values in a time window of −60–60 ms around these lags (computed in 20-ms steps).

The first question of this study as outlined in the introduction concerns the tracking (MI) of individual feature groups in temporal and occipital ROIs and the two experimental conditions, respectively, and more so if a given ROI reflects a given feature (e.g., the unheard acoustic envelope) independently of the physically present other feature (e.g., the visible lip movements in the visual-only condition). To quantify whether the tracking of each feature group (in a given ROI and frequency band) is statistically redundant with (or possibly complementary to) the other group, we calculated the conditional MI (CMI) between MEG and one feature group, partialling out the respective other group (Fig. 3, CMI values; Giordano et al., 2017; Ince et al., 2017). Specifically, the CMI measure allows us to quantify the unique information shared between a variable and the MEG while controlling for the information provided by the conditional variable. Mathematically, it can be described as the following:

I(X;Y|Z) = H(X,Z) + H(Y,Z) − H(X,Y,Z) − H(Z),

where *I* denotes the CMI and *H* the joint entropies between combinations of variables *X*, *Y* and the conditional variable *Z*. Similarly, we also determined the CMI between the MEG and each individual feature, obtained by partialling out all other visual and auditory features (Fig. 4). To be able to compare the MI and CMI estimates directly, we ensured that both estimates had comparable statistical biases. To achieve this, we effectively derived the MI as a conditional estimate, in which we partialled out a statistically-unrelated variable. That is, we defined

MI(feature ; MEG) ≅ MI(feature ; MEG|time_shifted_feature).

Here, time_shifted_feature is a representation of the respective feature(s) with a random time lag and hence no expected causal relation to the MEG. Each MI estimate was obtained by averaging this estimate over 2000 repetitions of a randomly generated time-shifted feature vector. To render the (conditional) MI estimates meaningful relative to the expectation of zero MI between MEG and stimulus features, we furthermore subtracted an estimate of the null-baseline of no systematic relation between signals. This was obtained by computing (conditional) MI values after randomly time-shifting the stimulus feature(s) and averaging the resulting surrogate MI estimates over 100 randomizations.

### Relating MI to word recognition performance

The behavioral performance for each participant and condition was obtained as the percent correctly (PC) reported target words (obtained in a four-choice task). To probe the second question of whether the tracking of restored features relates to word recognition performance we relied on partial regression. Specifically, we probed the linear relation of word recognition performance and feature tracking across participants while accounting for potential spurious correlations between these because of variations in the individual signal-to-noise ratio in each participants’ MEG data. We predicted the PC in the visual-only trials based on (1) the individual MI for aud env in the temporal ROI and the MI for aud pitch in the occipital ROI as the primary variables of interest, (2) the tracking of LipFeat (MI) in the occipital ROI in visual trials, and (3) the tracking of AudFeat in the temporal ROI in auditory trials. The last two serve as potentially confounding variables, as they provide a proxy to the overall SNR of the speech and lip tracking in the respective dataset. By focusing on aud env/aud pitch in the temporal/occipital ROIs respectively, we predicted task performance based on the individual features that were most associated with the tracking of AudFeat (compare Fig. 4*C*,*D*). To establish these regression models, we z-scored the MI values of interest (variables 1–3) and the PC across participants. For the confounding variables, we applied the z-scoring for each frequency band and subsequently averaged the z-scored values across bands. For each frequency band, we created a single model containing all target and confounding variables. From the respective models we obtained the significance of each predictor of interest. Furthermore, we compared the predictive power of this full model with that of a reduced model not featuring the predictors of interest (variable 1). From the likelihoods of each model, we derived the relative Bayes factor (BF) between these based on the respective BIC values obtained from each model. For visualization we used partial residual plots using the procedure described by Velleman and Welsch (Velleman and Welsch, 1981). This procedure was applied to each individual feature of interest (i.e., aud env and aud pitch).

### Statistical analysis

Statistical testing of MI data was based on a nonparametric randomization approach incorporating corrections for multiple comparisons (Nichols and Holmes, 2003). To test whether the group-level median MI (or CMI) values were significantly higher than expected based on the null hypothesis of no systematic temporal relation between sensory features and MEG, we proceeded in a similar fashion as in previous work (Giordano et al., 2017; Bröhl and Kayser, 2021): we obtained a distribution of 2000 MI values between randomly time-shifted MEG and the stimulus vectors, while keeping the temporal relation of individual features to each other constant. This distribution was obtained for each participant, frequency band, feature group (AudFeat and LipFeat), ROI (temporal, occipital), and condition (A-only, V-only) separately. To correct for multiple comparisons, we generated a single random distribution by pooling the randomly generated MI values across all dimensions except frequency bands, given that the MI values decreased considerably across bands (Fig. 3), and selecting the maximum 2000 values, thereby creating a random maximum null distribution (Nichols and Holmes, 2003). We then tested the group-level median against the 99th percentile of this maximum distribution as a significance threshold, which effectively implements a one-sided randomization test at *p* < 0.01 corrected for all dimensions except frequency bands. To test for differences between MI and CMI values for a given condition, band and ROI, we also used a permutation approach combined with a Wilcoxon signed-rank test; first, we established the respective true Wilcoxon z-statistic between MI and CMI values; then we created a distribution of surrogate z-statistics under the null hypothesis of no systematic group-level effect, obtained by randomly permuting the labels of MI and CMI values 5000 times. From this, we obtained the maximum across features, bands, ROIs and conditions to correct for multiple comparisons and used the 99th percentile of this randomization distribution to determine the significance of individual tests.

The CMI values for individual features in Figure 4 were compared using a one-way repeated measure Kruskal–Wallis rank test, followed by a *post hoc* Tukey–Kramer multiple comparison. We used the same procedure to test for differences between CMI values in the subareas composing each ROI (Table 1). To test CMI values between hemispheres, we used a Wilcoxon signed-rank test (Table 2). The resulting *p*-values were corrected for false discovery rate using the Benjamini–Hochberg procedure within each set of comparisons (Benjamini and Hochberg, 1995). In all tests, an α level of α < 0.01 was deemed significant. For all statistical tests we provide exact *p*-values, except for randomization tests where the approximate *p*-values were smaller than the inverse of the number of randomizations.

**Table 1:** Feature tracking in individual anatomic areas within temporal and occipital ROIs

		0.5–1 Hz				1–3 Hz			
ROI	Anatomical area	AudCMI	Chisq; *p*val	LipCMI	Chisq; *p*val	AudCMI	Chisq; *p*val	LipCMI	Chisq; *p*val
A-only trials Temporal	A41/42	0.97	**27.02; 4.7e-05**	0.096	2.47; 0.59	0.19	**29.62; 2.7e-05**	0.032	5.14; 0.32
	TE1.0/1.2	0.56		0.099		0.11		0.028	
	A22c	0.86		0.098		0.18		0.033	
	A22r	0.5		0.093		0.095		0.029	
Occipital	mOccG	0.1	3.50; 0.47	0.073	0.66; 0.88	0.025	2.71; 0.58	0.02	1.97; 0.66
	OPC	0.09		0.069		0.025		0.02	
	iOccG	0.11		0.068		0.027		0.021	
	msOccG	0.11		0.074		0.029		0.021	
V-only trials Temporal	A41/42	0.33	4.59; 0.36	0.1	5.14; 0.32	0.034	5.24; 0.32	0.027	1.00; 0.85
	TE1.0/1.2	0.25		0.088		0.031		0.028	
	A22c	0.34		0.1		0.035		0.027	
	A22r	0.22		0.085		0.028		0.029	
Occipital	mOccG	0.13	3.90; 0.44	0.17	12.30; 0.026	0.045	8.20; 0.13	0.15	14.57; 0.012
	OPC	0.14		0.19		0.06		0.2	
	iOccG	0.14		0.2		0.048		0.17	
	msOccG	0.11		0.11		0.039		0.082	

The table lists CMI values of either set of features (AudCMI, LipCMI) and a statistical comparison between the individual atlas-defined areas of the temporal and occipital ROIs [Kruskal–Wallis tests, reporting chi-squares (Chisq) and *p*-values (*p*val)]. Bold numbers indicate statistically significant results. *P*-values are FDR-corrected within this table.

**Table 2:** Feature tracking in each hemisphere

		0.5–1 Hz				1–3 Hz			
ROI	Hemisphere	AudCMI	z; *p*val	LipCMI	z; *p*val	AudCMI	z; *p*val	LipCMI	z; *p*val
A-only trials Temporal	Left	0.8	1.20; 0.59	0.094	−0.33; 0.74	0.13	−0.81; 0.59	0.028	−1.11; 0.59
	Right	0.64		0.099		0.15		0.031	
Occipital	Left	0.1	−0.37; 0.74	0.07	−0.33; 0.74	0.027	0.81; 0.59	0.021	0.63; 0.65
	Right	0.1		0.072		0.025		0.02	
V-only trials Temporal	Left	0.31	0.89; 0.59	0.098	0.76; 0.59	0.035	0.85; 0.59	0.025	−1.85; 0.26
	Right	0.26		0.091		0.03		0.03	
Occipital	Left	0.11	−2.24; 0.2	0.14	−2.98; 0.046	0.043	−1.68; 0.3	0.13	−2.07; 0.21
	Right	0.15		0.2		0.054		0.18	

The table lists CMI values of either set of features (AudCMI, LipCMI) and a statistical comparison between hemispheres of each ROI [Wilcoxon signed-rank tests, reporting z values (z) and *p*-values (*p*val)]. *P*-values are FDR-corrected within this table.

### Data and code availability

Data and code used in this study are publicly available on the Data Server of the University of Bielefeld (https://gitlab.ub.uni-bielefeld.de/felix.broehl/fb02).

## Results

### Acoustic and visual features are tracked in temporal and occipital cortices

Participants were presented with either spoken speech (in A-only trials) or a silent video of the speaking face (in V-only trials) and were asked to report a target word for each sentence in a four-choice word recognition task. The behavioral data show that participants were well able to detect the correct word both during acoustic speech embedded in noise and during lip reading and achieved overall similar levels of performance in both conditions (Fig. 1*B*, median fraction correct responses for A-only = 0.7, V-only = 0.71; *n* = 18). To quantify the tracking of relevant features, we defined three auditory (AudFeat) and three visual (LipFeat) features respectively based on the acoustic waveform and the lip trajectory (Fig. 1*A*). An analysis of their temporal coherences revealed that they were coherent in the frequency bands of interest (e.g., 1–3 Hz, envelope-lip area coherence of ∼0.2; Fig. 1*D*). The overall pattern of coherence and the degree of temporal relation between acoustic features and lip movements in the present material is comparable with those in other datasets (Chandrasekaran et al., 2009; Park et al., 2016; Giordano et al., 2017; Hauswald et al., 2018).

Previous work has shown that in the dataset analyzed here temporal and occipital brain regions reflect auditory and visual speech signals respectively (Keitel et al., 2020). We extend this observation to the entire group of acoustic (AudFeat; Fig. 2*A*) or lip features (LipFeat; Fig. 2*B*) using a MI approach. The whole-brain maps show the expected prevalence of acoustic (visual) tracking in temporal (occipital) regions. Given that our main questions concerned the tracking of features specifically in occipital and temporal brain regions, we focused the subsequent work on atlas-based ROIs (Fig. 2*C*; the temporal ROI shaded in mint and the occipital ROI shaded in purple; for details, see Materials and Methods).

### Temporal and occipital cortex represent acoustic speech features during silent lip reading

To address the question of whether temporal and occipital cortices represent auditory and visual speech features during lip reading, we performed a comprehensive analysis of the tracking of both sets of features across a range of frequency bands during auditory (A-only) and visual (V-only) conditions (MI values; Fig. 3). To further quantify whether the tracking of each feature group is possibly redundant with the tracking of the respective other feature group, we derived CMI values for each feature group, obtained by partialling out the respective other group (CMI values). By comparing MI and CMI values we can test, for example, whether the temporal ROI tracks the unheard speech envelope during silent lip reading also when discounting for the actually presented lip trajectory. In the following we discuss the results per sensory modality and ROI.

As expected, when listening to speech (A-only), the temporal ROI significantly tracks auditory features (AudFeat) in all frequency bands tested (Fig. 3, top row, red MI data; nonparametric randomization test, all bands: *p* < 5 × 10^−5^). This tracking persists when discounting potential contributions of the not-seen visual features (red CMI data all individually significant: *p* < 5 × 10^−5^), though in some bands the CMI values were significantly lower than the unconditional MI (Wilcoxon signed-rank test comparing MI vs CMI, 2–4 Hz: z = 3.59, 3–6 Hz: z = 3.68, 4–8 Hz: z = 3.42, all comparisons: *p* < 2 × 10^−5^). During the same auditory trials, lip features are only marginally reflected in the temporal ROI, as shown by low but significant MI and CMI values above 1 Hz (Fig. 3, top row, cyan MI and CMI data; all bands above 1 Hz: *p* < 5 × 10^−5^). This tracking of visual features was significantly reduced when partialling out the physically presented auditory features (2–4 Hz: z = 3.59, 3–6 Hz: z = 3.68, 4–8 Hz: z = 3.42, all comparisons: *p* < 2 × 10^−5^).

During lip reading (V-only), the temporal ROI tracks the unheard auditory features, particularly below 1 Hz (Fig. 3, second row, red MI data; all bands: *p* < 5 × 10^−5^). Except in the 2-4 Hz range, the temporal ROI tracks the unheard AudFeat to a similar degree as when discounting the actually presented visual signal (significant red CMI values, all bands: *p* < 5 × 10^−5^) as there were no significant differences between MI and CMI values except one band (2–4 Hz: z = 3.42, *p* < 1 × 10^−4^, see asterisks). The physically presented lip movements during these V-only trials were also tracked significantly in the temporal ROI (Fig. 3, second row; cyan MI and CMI data, 1–6 Hz: *p* < 5 × 10^−5^) but the CMI values were only marginally above chance level, suggesting that genuine visual representations in the temporal region is weak.

As expected, during lip reading (V-only), the occipital ROI tracks lip features (LipFeat) across frequency bands (Fig. 3, bottom row, cyan MI values; all bands: *p* < 5 × 10^−5^). Again, this tracking persists after partialling out the nonpresented acoustic features (cyan CMI values; all bands: *p* < 5 × 10^−5^), although the CMI values were significantly lower than the MI (all bands above 1 Hz: z ≥ 3.72, *p* < 2 × 10^−5^). This indicates some redundancy between the tracking of the physically present lip trajectory and that of the unheard auditory features. Confirming this, occipital tracking of the physically presented lip signals emerges in parallel with that of the nonpresented auditory features (Fig. 3, bottom panel, red MI data; all bands: *p* < 5 × 10^−5^). This occipital tracking of unheard auditory features was significantly reduced when partialling out the lip signal (MI vs CMI data; all bands above 1 Hz: z ≥ 3.72, *p* < 2 × 10^−5^) but remained statistically significant (red CMI data; all bands: *p* < 5 × 10^−5^).

Finally, when listening to speech (A-only), the occipital ROI shows significant but weak tracking of auditory (Fig. 3, third row, red MI data; 1–6 Hz: *p* < 5 × 10^−5^) and visual features (cyan MI data; only 3–6 Hz: *p* < 5 × 10^−5^), suggesting that purely acoustic signals have a weak influence on the occipital brain region.

Collectively, these results show the expected representations of auditory features in temporal cortex during listening to speech and of lip features in occipital cortex during lip reading. In addition, they reveal that during lip reading, both temporal and occipital regions represent unheard auditory features and do so independently of co-existing representations of the physically presented lip movements. In the auditory cortex, this “restoration” of auditory signals prevails in the low delta band (0.5–1 Hz); in the visual cortex, this emerges in multiple bands.

To obtain an estimate of the effect size of the restoration of the unheard AudFeat during lip reading, we expressed the respective CMI values relative to those of the tracking of the respectively modality-preferred inputs of each ROI (Fig. 4*A*,*B*): for the temporal region the tracking of AudFeat during A-only trials and for the occipital region the tracking of LipFeat during V-only trials. In the temporal ROI, the restoration effect size, i.e., the tracking of AudFeat during lip reading, was about a third as strong as this feature’s tracking while directly listening to speech (Fig. 4*A*; AudFeat_V-only_/AudFeat_A-only_; 0.5–1 Hz: median = 0.37, 1–3 Hz: median = 0.24). In the occipital ROI, the tracking of AudFeat was about half as strong or stronger compared with the tracking of lip features when seeing the speaker (Fig. 4*B*; AudFeat_V-only_/LipFeat_V-only_; 0.5–1 Hz: median = 0.84, 1–3 Hz: median = 0.4). Albeit smaller than the tracking of the respective modality-preferred sensory inputs, the restoration of unheard auditory features still results in a prominent signature in temporally aligned brain activity in both cortices.

### Feature tracking is bilateral and prevails across anatomic brain areas

Having established the tracking of auditory and lip features in both temporal and occipital ROIs, we probed whether this tracking is possibly lateralized in a statistical sense and whether it potentially differs among the individual anatomic areas grouped into temporal and occipital ROIs respectively. While these analyses do not directly concern our main hypotheses outlined in the introduction, the issue of lateralization is pervasive in the literature on speech, and hence is addressed here for the sake of completeness. For this analysis we focused on the conditional tracking of each feature group. Comparing CMI values among anatomic areas (averaged across hemispheres) for each ROI (occipital, temporal), frequency band (0.5–1 and 1–3 Hz), condition and feature group revealed a significant effect of area for AudFeat tracking in the temporal ROI during A-only trials (Table 1; 0.5–1 Hz: χ^2^(3) = 27.02, *p* = 4.7 × 10^−6^, ε^2^ = 0.35; 1–3 Hz: χ^2^(3) = 29.62, *p* = 2.7 × 10^−6^, ε^2^ = 0.39; *p*-values FDR-corrected). *Post hoc* comparisons revealed that in both bands, tracking of AudFeat was higher in A41/42 and A22c compared with TE1.0/1.2 and A22r (Tukey–Kramer test, all tests *p* < 10^−5^). The effect of Area was close to but not significant for LipFeat tracking in the occipital ROI during V-only trials (0.5–1 Hz: χ^2^(3) = 12.3, *p* = 0.026, ε^2^ = 0.14; 1–3 Hz: χ^2^(3) = 14.57, *p* = 0.012, ε^2^ = 0.17). Importantly, these results suggest that while the tracking of auditory features was stronger in the early auditory region during A-only trials, the restoration of unheard auditory features during lip reading emerges to a similar degree among the individual temporal and occipital areas.

We performed a similar analysis comparing the CMI values within temporal or occipital ROIs between hemispheres. This revealed no significant effect of hemisphere (Table 2), hence offering no evidence for a statistical lateralization of feature tracking in the present data.

### Occipital cortex reflects pitch more than other acoustic features during lip reading

Having established that occipital and temporal regions track unheard auditory features, we then asked how individual features contribute to these representations. For this we focused on the following condition: the tracking of AudFeat in the delta range in V-only trials (Fig. 4*C*,*D*). We quantified the CMI for each individual feature, while discounting the evidence about all other left-out visual and auditory features, hence focusing on the unique tracking of each individual acoustic feature.

For the temporal ROI this revealed the prominent tracking of aud env (Fig. 4*C*). In the 0.5-1 Hz band only the CMI for aud env was above chance (*p* < 5 × 10^−5^) and there was a significant effect of feature (Kruskal–Wallis rank test χ^2^(2) = 9.27, *p* = 9.1 × 10^−4^, ε^2^ = 0.14). *Post hoc* tests revealed that the CMI for aud env differed significantly from that of aud slope (Tukey–Kramer test, *p* = 6.2 × 10^−4^; the other comparisons were not significant; *p* = 0.35 for env vs slope and *p* = 0.22 for slope vs pitch). In the 1-3 Hz band, the tracking of all auditory features was significant (all features: *p* < 5 × 10^−5^) and there was no significant effect of features (χ^2^(2) = 4.14, *p* = 0.13, ε^2^ = 0.04).

For the occipital ROI, this revealed a dominance of aud pitch (Fig. 4*D*). In the 0.5-1 Hz band, only the CMI of aud pitch was above chance (*p* < 5 × 10^−5^), a direct comparison revealed a significant effect of features (0.5–1 Hz: χ^2^(2) = 18.28, *p* = 1.07 × 10^−4^, ε^2^ = 0.32) and *post hoc* tests revealed a significant difference between aud pitch and aud slope (*p* = 7.03 × 10^−5^), while the other comparisons were not significant (*p* = 0.26 for pitch vs env and *p* = 0.02 for env vs slope). In the 1-3 Hz range, the tracking of all features was significant (all features: *p* < 5 × 10^−5^), there was a significant effect of feature (χ^2^(2) = 19.2, *p* = 6.77 × 10^−5^, ε^2^ = 0.34), and *post hoc* tests revealed a significant difference between pitch and slope (*p* = 3.61 × 10^−5^), while the other comparisons were not significant (*p* = 0.05 for pitch vs env and *p* = 0.12 for env vs slope). Collectively these results suggest that the restoration of acoustic signals in the occipital region emphasizes spectral pitch, while in the temporal region this emphasizes the temporal speech envelope.

### Tracking of auditory features is associated with lip-reading performance

Finally, we probed the second main question of whether the restoration of unheard auditory features during silent lip reading relates to word recognition performance. For this, we probed the predictive power of the MI about specific auditory features in either ROI for word recognition performance during V-only trials (Fig. 5). We specifically focused on the tracking of aud env in the temporal ROI and of aud pitch in the occipital ROI as the dominant feature-specific representations (compare Fig. 4*C*,*D*). Using linear models, we predicted word recognition scores across participants based on the tracking indices of interest and while discounting for potential confounds from differences in signal-to-noise ratio in the MEG data.

The results show that variations in word recognition scores are well predicted by the collective measures of feature tracking (0.5–1 Hz: *R*^2^ = 0.74, 1–3 Hz: *R*^2^ = 0.8). Importantly, the tracking of aud env in the temporal ROI was significantly predictive of lip-reading performance (Fig. 5, 0.5–1 Hz: β = 0.6, *p* = 0.037; 1–3 Hz: aud env β = 0.6, *p* = 2.8 × 10^−4^), while tracking of pitch in the occipital ROI was not (0.5–1 Hz: β = −0.13, *p* = 0.56; 1–3 Hz: β = −0.026, *p* = 0.91). This conclusion is also supported by BFs for the added predictive power of aud env and aud pitch to these models (aud env in the temporal ROI; 0.5–1 Hz: BF = 3.12; 1–3 Hz: BF = 26.34; aud pitch in the occipital ROI; 0.5–1 Hz BF = 0.3; 1–3 Hz BF = 0.24).

## Discussion

Natural face-to-face speech is intrinsically multidimensional and provides the auditory and visual pathways with partly distinct acoustic and visual information. These pathways could in principle focus mainly on the processing of their modality-specific signals, effectively keeping the two input modalities largely separated. Yet, many studies highlight the intricate multisensory nature of speech-related representations in the brain, including multisensory convergence at early stages of the hierarchy (Schroeder et al., 2008; Schroeder and Lakatos, 2009; Bernstein and Liebenthal, 2014; Crosse et al., 2015) as well as in classically amodal speech regions (Scott, 2019; Keitel et al., 2020; Mégevand et al., 2020). However, as the present results suggest, the auditory and visual pathways are also capable of apparent “restoring” information about an absent modality-specific speech component; while seeing a silent speaker, both auditory and visual cortices track the temporal dynamics of the speech envelope and the pitch contour respectively, in a manner that is independent on the physically visible lip movements. These “restored” representations of acoustic features relate to participants’ word recognition, suggesting that they may form a central component of silent lip reading.

### Auditory and visual cortex reflect acoustic speech features during lip reading

We systematically quantified the tracking of auditory and visual speech features during unisensory auditory and visual (lip reading) conditions in dynamically entrained brain activity. As expected, this confirmed that early auditory and visual regions reflect acoustic and visual features respectively at the time scales of delta (<4 Hz) and theta (4–8 Hz) band activity, in line with previous work (Aiken and Picton, 2008; Giraud and Poeppel, 2012; Doelling et al., 2014; Haegens and Zion Golumbic, 2018; Obleser and Kayser, 2019; Bauer et al., 2020). In addition, we found that during lip reading both regions contained significant information about unheard auditory features, also when discounting for the physically presented lip movements. This representation of acoustic features prevailed in low delta in auditory and delta and theta bands in visual cortex. Interestingly, this representation emphasized the temporal speech envelope in auditory cortex and spectral pitch in visual cortex. These results not only support that both regions are active during lip reading (Calvert et al., 1997; Ludman et al., 2000; Calvert and Campbell, 2003; Besle et al., 2008; Luo et al., 2010), but directly show that they contain temporally and feature-specific representations derived from lip movements that are relevant for comprehension.

These results advance our understanding of how the brain exploits lip movements in a number of ways. The restoration of auditory features during silent lip reading has been suggested in previous studies, one quantifying the coherence of temporal brain activity with the nonpresented speech envelope (Bourguignon et al., 2020) and others quantifying the coherence between occipital activity and the envelope (Hauswald et al., 2018; Suess et al., 2022). Yet, these studies differed in their precise experimental designs, their statistical procedures revealing the “restoration” effect, and did not probe a direct link to behavioral performance. The present data demonstrate that such tracking of auditory speech-derived features indeed emerges in parallel and in the same participants. Our data reveal the restoration of unheard acoustic features also when discounting the physically present lip signals (i.e., when using CMI). This finding is important, as the mere coherence of brain activity with the acoustic speech envelope may otherwise simply reflect amodal information contained in the physically-present visual speech that is directly redundant with the acoustic domain (Daube et al., 2019).

Furthermore, they show that this effect is largely bilateral and emerges across a number of anatomically-identified areas, suggesting that it forms a generic property of the respective pathways. Interestingly, the unheard auditory features during V-only trials were restored dominantly in the lower frequencies (0.5–3 Hz), similarly to recent results (Bourguignon et al., 2020). In principle, activity at these slow timescales may possibly reflect oro-facial cues such as head, eye or eyebrow movements (Munhall et al., 2004; Schroeder et al., 2008). We aimed to mitigate such confounds by instructing the speaker to move their head as little as possible and to avoid gestures, and by instructing participants to focus their gaze on the speaker’s lips. Moreover, our results align with recent work showing the restoration of the unheard acoustic envelope even when controlling for the speaker’s movement during visual presentation (Bourguignon et al., 2020). One may speculate whether this restoration reflects the synthesis of speech-specific elements. However, linguistic elements at this time scale mostly encompass phrasal structures, prosody or speech rhythm (Gross et al., 2013; Meyer et al., 2017; Keitel et al., 2018) and few of these are probably restored during lip reading in detail. Possibly, the restoration of the unheard envelope based on lip movements reflects processes for the temporal segmentation of speech-related information based on low-frequency activity (Doelling et al., 2014; Ghitza, 2017; Nidiffer et al., 2021).

These results come with an important caveat: the capability to read from lips alone is generally low in naive listeners (Grant and Seitz, 2000; Altieri et al., 2011), which poses an intricate problem when studying the cerebral basis of lip reading. To solicit a sufficient number of trials with successful lip reading and to balance word recognition performance between visual-only and auditory-only trials, we relied on a specifically designed experimental paradigm with two critical features. First, this paradigm relied on sentences constructed based on a repeating set of linguistic elements and a forced-choice task with a closed set of options. This limits the generalizability of the results toward naturally-produced everyday speech, as participants could in principle learn the mapping of only target words onto lip movements and choose the most likely one during the course of the experiment. Although we did not strictly control for this, both the chosen elements in each sentence as well as the target and distractor words were chosen randomly. Second, to familiarize participants with the material, the A-only condition preceded the V-only condition during the experiment. This may allow for memory-related processes to contribute to the observed restoration effects. However, the use of 180 syntactically similar but unique sentences makes it in our view highly unlikely that participants solely relied on the stimulus repetition and memory to solve the word recognition task. Rather, we believe that the restoration in the occipital cortex reflects the active parsing of the lip movement signal and engages specific visuo-phonetic transformations, as speculated previously (Hauswald et al., 2018; Nidiffer et al., 2021). This poses a possible solution to how the brain finds the best match between visually perceived speech and a word from a limited set of options. Nevertheless, the visual system might be primed to perceive visual speech after being familiarized with the underlying acoustic stimulus in a previous condition. Even in naturalistic listening situations one is likely to do so when observing a moving face. Therefore, we do not expect any priming of the visual system to confound the nature of lipreading in this paradigm compared to real-life situations. More so, this alludes to the origin of speech-related information during lip reading in general, as bottom-up processes may be aided by sentence-level predictions or expectations that contribute in a top-down manner and partially predict acoustic and lexical information based on the immediately preceding material (Cope et al., 2017). Given that lip-reading performance was higher than in other studies or real-life circumstances (Grant and Seitz, 2000; Altieri et al., 2011), it is possible that top-down processes exerted a stronger influence on early visual and auditory cortices in these data compared with real-life circumstances.

### Lip reading activates a network of occipital and temporal regions

Previous work has shown that lip movements activate a network of temporal, parietal, and frontal regions (Calvert et al., 1997; Paulesu et al., 2003; Pekkola et al., 2005; Capek et al., 2008; O’Sullivan et al., 2017; Ozker et al., 2018; Bourguignon et al., 2020) and that both occipital and motor regions can align their neural activity to the dynamics of lip movements (Park et al., 2016, 2018). The present data substantiate this, but also show that the representation of the physically visible lip trajectory along visual pathways is accompanied by the representation of spectral pitch, a type of selectivity not directly revealed previously (Suess et al., 2022). Spectral features are vital for a variety of listening tasks (Ding and Simon, 2013; Tivadar et al., 2018, 2020; Albouy et al., 2020; Bröhl and Kayser, 2021), and oro-facial movements provide concise information about the spectral domain. Importantly, seeing the speaker’s mouth allows discriminating formant frequencies and provides a comprehension benefit particularly when spectral features are degraded in the underlying acoustic speech (Plass et al., 2020). This suggests a direct and comprehension-relevant link between the dynamics of the lip contour and spectral speech features (Campbell, 2008). Hence, a representation of acoustic features during silent lip reading may underlie the mapping of lip movements onto phonological units such as visemes, a form of language-specific representation emerging along visual pathways (O’Sullivan et al., 2017; Nidiffer et al., 2021). This emphasizes the role of the visual system as an active agent during audiovisual speech processing.

Our results corroborate the notion that multisensory speech reception is not contingent only on high-level and amodal representations. Rather, they suggest that the brain likely exploits cross-modal correspondences between auditory and visual speech along a number of dimensions, including basic temporal properties (Chandrasekaran et al., 2009; Bizley et al., 2016) as well as mid-level features, such as pitch or visual object features, whose representation is traditionally considered to be modality specific (Schroeder et al., 2008; Zion Golumbic et al., 2013; Crosse et al., 2015; Plass et al., 2020). Previous work has debated whether visual speech is mainly encoded along the auditory pathways or whether occipital regions contribute genuine speech-specific representations (O’Sullivan et al., 2017; Ozker et al., 2018). Our results speak in favor of occipital regions supporting speech reception by establishing multiple forms of speech-related information, including those aligned with the acoustic domain revealed here, and those establishing visemic categories based on complementary visual signals (Nidiffer et al., 2021; Suess et al., 2022). Which precise occipital areas and by which patterns of connectivity they contribute to comprehension remains to be investigated, but both kinds of representations may well emerge from distinct temporal-occipital networks (Bernstein and Liebenthal, 2014). While visemic information may be driven by object-related lateral occipital regions, the more auditory-aligned representations such as the restoration of spectral signatures may be directly driven by the connectivity between occipital areas and superior temporal regions, which play a key role for audiovisual speech integration (Arnal et al., 2009; Lazard and Giraud, 2017). In the auditory cortex, the alignment of neural activity to the unheard speech envelope may reflect the predictive influence of visual signals on guiding the excitability of auditory pathways via low frequency oscillations (Schroeder et al., 2008). This alignment of auditory cortical activity to attended or expected sounds is well documented and has been proposed as a cornerstone of multisensory speech integration in general (Lakatos et al., 2008; Schroeder and Lakatos, 2009; Stefanics et al., 2010), and as shown here, directly relates to participants comprehension performance.

## Synthesis

Reviewing Editor: Christine Portfors, Washington State University Decisions are customarily a result of the Reviewing Editor and the peer reviewers coming together and discussing their recommendations until a consensus is reached. When revisions are invited, a fact-based synthesis statement explaining their decision and outlining what is needed to prepare a revision will be listed below. The following reviewer(s) agreed to reveal their identity: NONE. Note: If this manuscript was transferred from JNeurosci and a decision was made to accept the manuscript without peer review, a brief statement to this effect will instead be what is listed below.

Thank you for thoroughly addressing the reviewers’ comments.
